# Convolutional Neural Networks–Based Image Analysis for the Detection and Quantification of Neutrophil Extracellular Traps

**DOI:** 10.3390/cells9020508

**Published:** 2020-02-24

**Authors:** Aneta Manda-Handzlik, Krzysztof Fiok, Adrianna Cieloch, Edyta Heropolitanska-Pliszka, Urszula Demkow

**Affiliations:** 1 Department of Laboratory Medicine and Clinical Immunology of Developmental Age, Medical University of Warsaw, Zwirki i Wigury 63a Street, 02-091 Warsaw, Poland; acieloch@wum.edu.pl (A.C.); urszula.demkow@uckwum.pl (U.D.); 2 Postgraduate School of Molecular Medicine, Medical University of Warsaw, Zwirki i Wigury 61 Street, 02-091 Warsaw, Poland; 3 Department of Industrial Engineering & Management Systems, University of Central Florida, 4000 Central Florida Blvd., P.O. BOX 162993, Orlando, FL 32816-2993, USA; krzysztof.fiok@gmail.com; 4 Department of Immunology, The Children’s Memorial Health Institute, Aleja Dzieci Polskich 20, 04-730 Warsaw, Poland; er.pliszka@wp.pl

**Keywords:** neutrophil extracellular traps (NETs) quantification, automatic image analysis, convolutional neural networks (CNN), mask R-CNN, neutrophils, chronic granulomatous disease, reactive nitrogen species, nitric oxide, peroxynitrite

## Abstract

Over a decade ago, the formation of neutrophil extracellular traps (NETs) was described as a novel mechanism employed by neutrophils to tackle infections. Currently applied methods for NETs release quantification are often limited by the use of unspecific dyes and technical difficulties. Therefore, we aimed to develop a fully automatic image processing method for the detection and quantification of NETs based on live imaging with the use of DNA-staining dyes. For this purpose, we adopted a recently proposed Convolutional Neural Network (CNN) model called Mask R-CNN. The adopted model detected objects with quality comparable to manual counting—Over 90% of detected cells were classified in the same manner as in manual labelling. Furthermore, the inhibitory effect of GW 311616A (neutrophil elastase inhibitor) on NETs release, observed microscopically, was confirmed with the use of the CNN model but not by extracellular DNA release measurement. We have demonstrated that a modern CNN model outperforms a widely used quantification method based on the measurement of DNA release and can be a valuable tool to quantitate the formation process of NETs.

## 1. Introduction

Neutrophils are the most abundant leukocytes in human blood, constituting the first line of defense against infecting pathogens. For decades, neutrophils were thought to fight microorganisms via two major mechanisms—Phagocytosis followed by intracellular degradation in an oxygen-dependent or oxygen-independent manner and degranulation, i.e., the release of granular content into phagosomes or extracellular space [1,2]. A discovery of a novel mechanism in which the neutrophil may tackle an infection—The release of neutrophil extracellular traps (NETs)—Caused a rapid increase in the interests among the scientific community in the antimicrobial functions of granulocytes [3]. NETs were first described by Brinkmann et al. in 2004 as extracellular structures released by activated neutrophils, composed of a DNA backbone ornamented with antimicrobial proteins, such as myeloperoxidase (MPO), neutrophil elastase (NE), cathepsin B, and histones. Due to their physical properties and presence of high concentrations of lytic proteins, NETs are suggested to act as efficient traps for immobilizing and neutralizing pathogens. The current consensus states that NETs can be released either by the cells undergoing lytic cell death (in a process called NETosis) or without the destruction of plasma membranes [4]. In the latter mechanism, activation of neutrophils results in the release of nuclear DNA after the fusion of DNA-containing vesicles with plasma membranes or the extrusion of mitochondrial DNA [5,6]. To date, a large body of evidence indicates that NETs can be formed in response to bacterial, fungal, viral, or parasitic infections. Furthermore, neutrophils release NETs upon activation of neutrophils by various cytokines or biologically relevant molecules/particles, e.g., interleukin-8, platelet-activating factor, and monosodium urate crystals [7]. Despite a clearly beneficial role as a physical barrier controlling the spreading of an infection, NETs have also their dark side—Increased NETs formation has been associated with multiple pathological conditions, including autoimmune diseases, diabetes, or cancer [7]. A growing list of NETs-associated diseases gives rise to intense research on molecular mechanisms governing the process of NETs formation. The results of these studies might contribute to the development of new strategies for managing conditions arising from improper NETs formation and/or degradation [8].

Quantification of NETs released by isolated neutrophils in vitro constitutes an indispensable yet problematic element of NET-related research [9]. Existing protocols rely predominantly on two methods: analysis of microscopic images or spectrofluorometric quantification of DNA released by activated neutrophils [10]. The major pitfall of spectrofluorometric measurements is the inability to distinguish between NETosis and other modes of cell death as a source of extracellular DNA. On the other hand, manual analysis of microscopic images is time-consuming, laborious, generates intra-observer variability and hampers comparisons of results between different laboratories [9]. Accordingly, several authors provided digital, semi- or fully automatic solutions to facilitate the quantitative analysis of microscopic images. Most of the methodologies described previously rely on an analysis of paraformaldehyde-fixed samples stained either solely with DNA-binding dyes or in combination with immunofluorescently labeled antibodies [11,12,13,14,15,16]. However, one should bear in mind that fixation procedure precludes differentiation between dead and viable cells [17]. Moreover, immune-histological processing frequently causes artefacts—For example, washing off the fragile structure of NETs [18]. An alternative technique based on a digital processing of microscopic live images of cells stained solely with DNA-binding dyes bypasses the necessity of extensive sample handling and may eliminate some of potential artifacts related to sample preparation [17,19]. Furthermore, it does not require the use of expensive reagents. For this approach, the state-of-the-art methods of quantifying NETs release described in references [17,19] are semi-automatic, require applying threshold-based algorithms, and differentiate resting neutrophils from NETs on the basis of a single parameter—The size of the object. Notably, recent advances in the field concern the use of multispectral imaging flow cytometry for quantitative, high throughput analysis of NETs formation. However, this method may underestimate NETs released in the late stages of this process and requires high-cost, sophisticated equipment, which makes it unlikely to become widely accessible in the near future [20,21].

Here, we aimed to develop an automatic image processing method for detection and quantification of NETs based on live imaging and machine learning algorithm. Our work is based on the concept of Convolutional Neural Networks (CNNs), the origins of which can be traced back to 1968, when Hubel et al. [22] observed that the visual cortex of a monkey contains numerous neurons that respond only to small regions of the visual field. This observation inspired further research on artificial neural networks [23], which later in 1998 allowed for an introduction of a first seven-layer CNN that achieved impressive performance on 32 × 32 pixel images [24]. With the availability of GPU computing machines that dramatically speeded-up calculations, in 2012, CNNs demonstrated their superiority over previously known methods in detection of objects in images [25]. As a consequence, more and more image analysis problems, including those with a profound impact on research in cell biology and infectious biology [26,27,28] are addressed with the use of CNNs. The actual limitation for new applications of this machine learning technique is the lack of training datasets required for a CNN model in the training stage, before the model can be used in the inference mode. Our contribution is creating a public dataset that can be used by other researchers to train CNN models for quantification of NETs based on live imaging. In order to demonstrate that at least near-human performance in detecting objects in microscopical images can be achieved with use of CNNs in this field, we adopted a recently proposed model called Mask R-CNN [29], carried out training, and evaluated the model on our dataset. We also present comparison of quantification carried out by the adopted CNN model and spectrofluorometry.

## 2. Materials and Methods

### 2.1. Procedures for Isolation and Stimulation of Neutrophils; Visualization of NETs

#### 2.1.1. Reagents

Roswell Park Memorial Institute (RPMI) 1640 medium, HEPES, micrococcal nuclease (MNase), Hoechst 33342, SYTOX Green and SYTOX Orange were purchased from Thermo Fisher Scientific (Waltham, MA, USA). Anti-NE (ab21595) and secondary anti-rabbit FITC-conjugated (ab6717) antibodies were purchased from Abcam (Cambridge, UK). Peroxynitrite and *S*-nitroso-*N*-acetyl-dl-penicillamine (SNAP) were purchased from Cayman Chemicals (Ann Arbor, MI, USA) and GW 311616A (neutrophil elastase inhibitor, NEi) was purchased from Axon Medchem (Groningen, The Netherlands). All other reagents, unless otherwise stated, were purchased from Sigma Aldrich (St. Louis, MO, USA).

#### 2.1.2. Isolation of Human Neutrophils and Stimulation of NETs Release

This study was approved by the Ethics Committee of the Medical University of Warsaw, Poland (reference numbers: KB/225/2014 and KB/55/A/2017), and the investigations were carried out following the rules of the Declaration of Helsinki. For most experiments, samples of peripheral blood of adult healthy blood donors were collected at and purchased from Regional Blood Donation Center. According to Polish legal regulations, each donor signed an informed written consent which enabled blood donation center to sell their blood constituents for research purposes. The blood was also collected from seven patients, including three children, suffering from chronic granulomatous disease (CGD; age in years mean ± SEM: 6 ± 1.7) and three control, healthy children (age in years, mean ± SEM: 6 ± 1.7). CGD diagnosis was based on clinical symptoms and the defective production of superoxide, as confirmed by nitroblue tetrazolium assay and/or dihydrorhodamine 123 oxidation assay by flow cytometry. Informed written consent was signed by each individual or their caretakers.

Neutrophils were isolated using density gradient centrifugation and a polyvinyl alcohol sedimentation method, as described elsewhere [30]. Isolated neutrophils were resuspended in RPMI 1640 without phenol red supplemented with 10 mM HEPES. Cells were seeded into wells of appropriate plates (as indicated below), pre-treated with NEi (20 μM) when necessary, and allowed to settle for 30 min. Subsequently, cells were stimulated with 100 nM phorbol 12-myristate 13-acetate (PMA), 500 μM SNAP (nitric oxide donor), or 100 μM peroxynitrite for 1–3 h at 37 °C, 5% CO_2_. Alternatively, culture medium (RPMI 1640 without phenol red supplemented with 10 mM HEPES) was added to the wells instead of NETs-inducing agents; these samples served as negative controls.

#### 2.1.3. Microscopic Live Imaging of NETs

To visualize in vitro NETs release, 2 × 10^4^ cells per well (1 × 10^5^ cells/mL) were seeded into 48-well plates and treated as stated above. Fifteen minutes before indicated time point, cells were simultaneously stained with Hoechst 33342 (1.25 μg/mL) to visualize nuclear DNA in all cells and SYTOX Green (100 nM) to visualize DNA of cells with compromised cell membranes. At indicated timepoint NETs were visualized using an inverted fluorescent microscope Leica DMi8 equipped with a 40× magnification objective. During each experiment, imaging parameters (exposure time and light intensity) were adjusted so that the fluorescence signal from the brightest objects in both channels was ≥235 arbitrary units (8-bit camera).

#### 2.1.4. Immunofluorescence Staining of NETs

Neutrophils were seeded into 8-well Lab-Tek chambers (2.5 × 10^4^ cells/well, 6.25 × 10^4^/mL) and stimulated as stated above. After a two-hour incubation, samples were fixed with 4% paraformaldehyde for 20 min, permeabilized with 0.1% Triton X-100, and blocked with 5% bovine serum albumin (BSA) and 10% goat serum. Samples were stained with antibodies directed against NE (1:100, overnight, 4 °C) and secondary antibodies conjugated with FITC (1:2000, 1 h, room temperature (RT). DNA was counterstained with 1 μM SYTOX Orange. NETs were visualized using an inverted fluorescent microscope Leica DMi8 equipped with a 40× magnification objective.

### 2.2. Open NETs Dataset for Image Analysis Models

**Figure 1:**
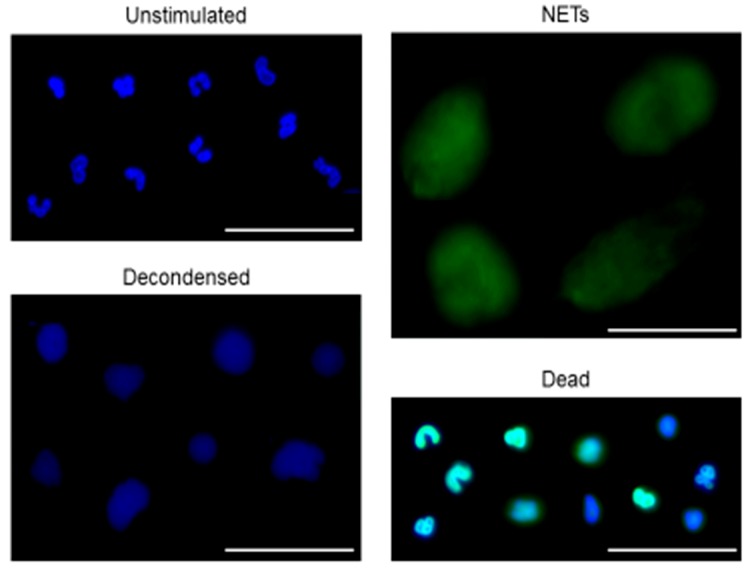
Representative examples of objects manually assigned as unstimulated, decondensed, neutrophil extracellular trap (NET)-releasing or dead cells. Isolated human neutrophils were seeded into plates, pre-incubated with or without neutrophil elastase inhibitor (NEi) for 30 min and stimulated with phorbol 12-myristate 13-acetate (PMA), *S*-nitroso-*N*-acetyl-dl-penicillamine (SNAP) or peroxynitrite or left unstimulated. After 1–3 hours of incubation, cells were simultaneously stained with Hoechst 33342 and SYTOX Green. Samples were visualized with inverted fluorescent microscope and 300 images were gathered at 40× magnification to create a NETs dataset. The observed objects were manually assigned into four categories: unstimulated, decondensed, NET-producing, and dead cells. In this figure representative examples of objects assigned into aforementioned categories are shown. Bar = 50 μm.

To advance research regarding quantification of NETs release, we have created a dataset of microscopical pictures of neutrophils stimulated to release NETs. In our studies we used live imaging approach and we make the dataset available to the public together with bounding box ground truth annotations. A dataset of microscopical pictures of neutrophils with bounding box ground truth annotations is available at https://github.com/krzysztoffiok/CNN-based-image-analysis-for-detection-and-quantification-of-neutrophil-extracellular-traps. All images were acquired according to the procedures described above and stained with SYTOX Green and Hoechst 33342. The objects in the images were manually assigned by one qualified scientist into four categories: unstimulated, decondensed, NET-producing and dead cells. Objects stained blue (solely with Hoechst 33342), of polymorphonuclear shape were classified as “unstimulated” cells; blue decondensed objects of round shape, bigger than unstimulated cells, were designated as “decondensed” cells. The presence of “decondensed cells” can be interpreted as an early stage of neutrophils activation preceding NETs release. Big, decondensed objects stained both with SYTOX Green and Hoechst 33342 of characteristic cloud-like or net-like appearance were designated as “NETs”. Objects stained with both DNA dyes, of condensed chromatin and smaller size than NETs, were classified as “dead” cells. Notably, this type of cells can be a source of extracellular DNA, which interferes with fluorometric measurements and falsely elevates the estimates of NETs release levels. Examples of objects classified into the aforementioned categories are shown in Figure 1.

Ground truth annotations were created with the use of open software [31] in popular Pascal VOC [32] format and are also provided in MS CoCo [33] format. The acquired images were randomly divided into training data set (61.6% images, 62.5% cells), validation data set (19.7% images, 21.5% cells), and test data set (18.7% images, 16% cells). Following random split of the images, the data sets consisted of

- Training data set: images from 15 individuals, 5–27 images (mean ± SEM: 12.5 ± 1.9) and 65–473 labeled objects (mean ± SEM: 210.6 ± 37.3) per each individual;
- Validation data set: images from four individuals, 2–22 images (mean ± SEM: 15.0 ± 4.5) and 27–438 labeled objects (mean ± SEM: 332.5 ± 101.8) per each individual;
- Test data set: images from six individuals, 6–12 images (mean ± SEM: 12.5 ± 1.0) and 122–271 labeled objects (mean ± SEM: 169.3 ± 22.8) per each individual.

Further characteristics of the dataset are presented in Table 1.

### 2.3. CNN Quantification Method

In our work, we applied a known Convolutional Neural Network (CNN) model to address the problem of quantification of NETs release. The choice of proper CNN architecture is connected with speed–quality tradeoff. In our application, the goal was to carry out a high-quality analysis of microscopic images that does not require real-time operation. Therefore, we adopted a model that is slow but provides high-quality results, namely Mask R-CNN [29]. For our work, we chose an open source implementation by Matterport [34] in Python with the use of Keras and Tensorflow frameworks. The model and its implementation has already gained popularity among various researchers [35,36,37,38,39,40] for of several reasons—The model is published under MIT license, which allows users to modify the model; it adopts well established CNN backbone ResNet [41] and recently introduced concepts like Feature Pyramid Network (FPN) [42] and ROI Align that in terms of quality make Mask R-CNN superior to comparable models like Faster R-CNN; the maximum accepted input image resolution of the model (1024 × 1024 pixels) is high when compared to many previously developed CNN models like YOLO (up to 608 × 608 pixels) [43] or Faster R-CNN [42] Python implementation (600 × 1000 pixels). The ability to analyze higher resolution images is important especially when dealing with small objects like biological cells [35].

#### 2.3.1. Model Training

Training of the adopted model was carried out on a single GPU Tesla K80 12 GB RAM, and the above described dataset was only slightly augmented for training by affine transformations. Due to limited hardware resources the whole training was carried out at batch size = 1. All hyper-parameters and training parameters are provided in the Supplementary Materials. We carried out transfer learning started from the MS CoCo pre-trained model weights. As a result, the later described performance of our model can be treated as a baseline that should be easy to outperform.

#### 2.3.2. Performance Metrics for the CNN Model

To assess model performance, subsequent metrics in the object detection domain were adopted: MS CoCo (Average Precision at 10 Intersection over Union (IoU) levels of 0.50:0.95) [33]; Pascal VOC (Average Precision and at IoU 0.50) [32]. In addition, we have also calculated the values of: Average Precision at IoU 0.10; Average Recall at IoU 0.50; Average Recall at 10 IoU levels 0.50:0.95; Average Recall at IoU 0.10.

The metrics are rarely calculated at low IoU levels, such as 0.10, due to the fact that this would imply that model accepts high localization error. We have decided to add this assessment parameters because for our research goal, which is simple quantification of the objects, their proper localization is of secondary importance. All of calculated parameters were averaged over four object categories. Also, we have followed the MS CoCo metrics approach and added distinction of parameter’s values according to the size of analyzed objects. The object’s sizes were divided into three groups—Smaller than 32 square pixels, medium between 32 and 96 square pixels, and larger than 96 square pixels.

### 2.4. Quantification by Measurement of Extracellular DNA Release

Neutrophils (5 × 10^4^ cells per well, 1.25 × 10^5^ cells/mL) were seeded into 48-well plates and stimulated as stated above. After 2 h incubation, 500 mIU of MNase were added to each well to detach DNA from the cell surface. Following a 20 min incubation, the activity of MNase was stopped with 5 mM EDTA, and the plates were centrifuged for 10 min at 415 g. Supernatant was then transferred into a black 96-well plate in triplicates, and 500 nM of SYTOX Green were added to each well. The amount of DNA released from the cells was measured in a FLUOstar OMEGA plate reader (BMG Labtech, Ortenberg, Germany).

The data were normalized and presented as the fold changes in NETs formation. Mean fluorescence readout from unstimulated cells was set as 100% DNA release in each donor. Subsequently, individual fluorescence readouts from samples stimulated with peroxynitrite and/or pretreated with NEi were normalized to this value and shown as the percentage.

### 2.5. Statistical Analysis

The results were analyzed using GraphPad Prism version 5 (one-way ANOVA) or 6 (two-way ANOVA). Kolmogorov-Smirnov test was used to check if the values come from a Gaussian distribution. Multiple groups were compared with a one-way ANOVA test with post-hoc Bonferroni’s multiple comparison test. Alternatively, two-way ANOVA with Tukey’s post hoc test was used. P ≤ 0.05 was considered as significant.

## 3. Results

### 3.1. The Adopted Model Detects Objects with Quality Comparable to Manual Counting

To create the NETs dataset, neutrophils isolated from healthy blood donors were stimulated with PMA, SNAP, and peroxynitrite for up to 3 h. When appropriate, cells were incubated with NEi for 30 min prior to stimulation. At the indicated timepoint, cells were simultaneously stained with Hoechst 33342 and SYTOX Green and visualized. As we and others reported previously [44,45,46], all three stimuli efficiently induced NETs release and preincubation with NEi diminished NETs formation (Supplementary Figure S1). NETs formation could be consecutively observed as the loss of lobulated nuclear shape, rounding of the nucleus and loosening of the chromatin structure followed by the rupture of cell membrane and release of cloud-like NETs structures. In SNAP- and PMA-stimulated samples pretreated with NEi, NEi activity was manifested mostly as a decrease in a fraction of cells releasing NETs and an increase in a fraction of multilobulated cells, stained only with Hoechst 33342. Conversely, samples preincubated with NEi and stimulated with peroxynitrite could be distinguished by the dominance of small, condensed object stained both with SYTOX Green and Hoechst 33342, classified as “dead” cells. Images of stimulated cells and negative controls were taken, and objects were manually assigned into four different categories and this dataset was used for training of the adopted CNN model as described in Methods.

To enlarge NETs dataset, we included similarly processed images of neutrophils isolated from CGD patients and stimulated with SNAP or peroxynitrite. As we reported previously, peroxynitrite efficiently stimulated NETs release, whilst no or very weak NETs release upon incubation with SNAP was observed [46] (Supplementary Figure S2).

**Figure 2:**
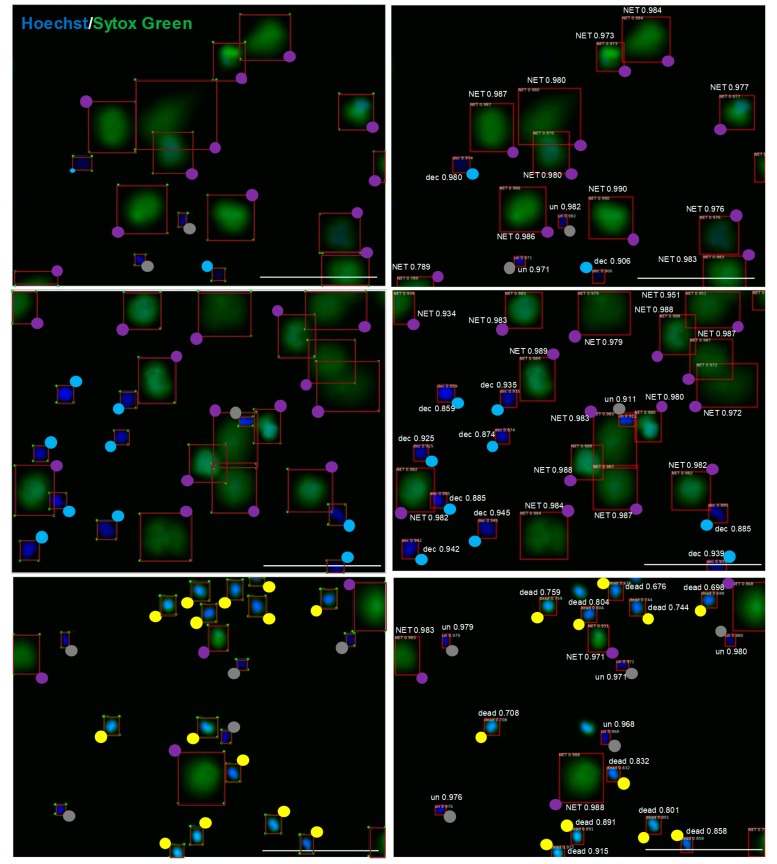
The adopted model trained on our preliminary dataset is able to detect objects with quality comparable to manual labeling. Images of unfixed neutrophils stained with Hoechst 33342 and SYTOX Green were analyzed by the CNN model (right) in parallel with manual analysis (left). To facilitate the assessment of labeling, colored dots are shown at one of the four vertexes of a rectangle surrounding the object. Grey dots are unstimulated cells (un), blue dots are decondensed cells (dec), violet dots are NETs, yellow dots are dead cells. Numerical values represent model’s confidence in the given cell class prediction—1 is maximum confidence, and the scale bar is 100 μm.

To validate the ability of the adopted model to detect and qualify cells into pre-determined categories, we prepared a set of 57 images containing almost 1000 objects not previously used to train our model. These images were subjected to automatic analysis using the trained CNN model, and the results of this analysis were compared with the results of manual analysis performed by a person who had previously prepared the dataset for training. When considering results with higher localization error (IoU = 0.5 and IoU = 0.1), the CNN model achieved average recall of 0.925 and 0.931 respectively, thus it was able to detect over 92% of all manually detected cells. At the same time, the average precision of the model was 0.906 and 0.913, respectively, meaning that over 90% of detected cells were classified in the same manner as in manual labelling. Detailed results of the comparison are presented in Table 2. Examples of detections made by the CNN model compared to manual human labeling are shown in Figure 2. The pre-trained CNN model acquired a good performance in detecting NETs that overlapped and/or consisted of highly decondensed chromatin (thus exhibiting less bright fluorescence than NETs covering smaller areas), yet these types of NETs were more likely to be missed during the automatic analysis than brightly-stained, well-separated NETs. Examples of NETs incorrectly classified by the pre-trained CNN model are shown in Supplementary Figure S3.

### 3.2. The CNN Model is Superior to NETs Quantification Based on Extracellular DNA Release

Next, we compared the performance of the CNN model with the widely used assessment of NETs formation based on the of measurement DNA release. Briefly, isolated neutrophils were pretreated with NEi for 30 min and stimulated with peroxynitrite for 2 h. At the indicated timepoint, NETs formation was assessed fluorometrically and simultaneously images of unfixed cells stained with Hoechst 33342 and SYTOX Green were taken, which were then both inspected visually by an operator and underwent analysis using the adopted model. Effect of NEi on NETs formation was also verified using immunofluorescent detection of NETs and manual microscopic evaluation.

**Figure 3:**
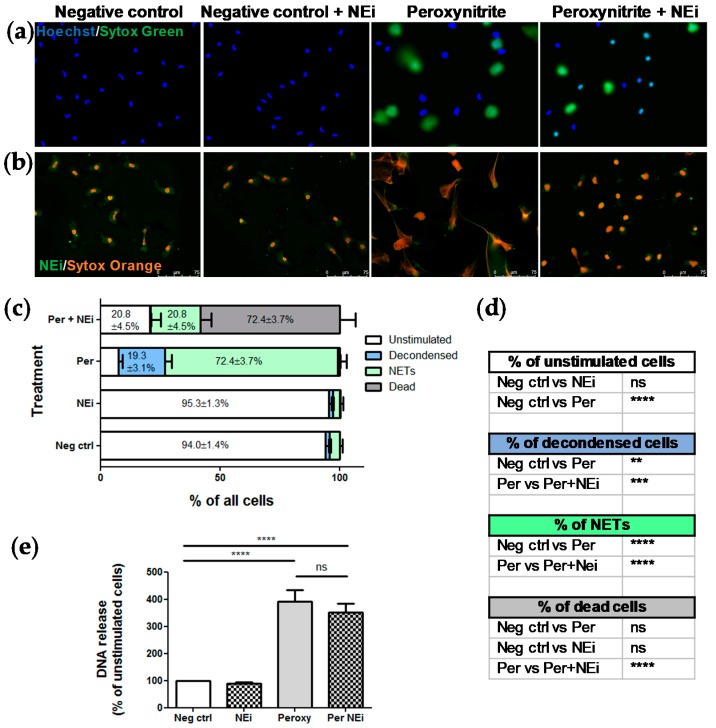
The adopted CNN model, contrary to DNA release measurement, confirmed the inhibitory effect of NEi on NETs release. Peripheral blood neutrophils were isolated from peripheral blood of six healthy donors, samples were pretreated with NEi for 30 min and/or stimulated with peroxynitrite for 2 h. Negative control—Unstimulated cells, incubated only with RPMI 1640. (**a**) Representative images of samples pretreated with NEi and/or stimulated with peroxynitrite using live imaging with Hoechst 33342 and SYTOX Green; (**b**) representative images of immunofluorescently-labeled samples pretreated with NEi and/or stimulated with peroxynitrite; (**c**, **d**) for each patient, 10 images of unfixed cells stained with Hoechst 33342 and SYTOX Green were taken per each one of four experimental conditions (negative control; cells pretreated with NEi; cells stimulated with peroxynitrite; cells pretreated with NEi and stimulated with peroxynitrite). The images were analyzed using the adopted model and the results were analyzed as percentage of objects of different classes and compared between groups. (**d**) n = 6, 2-way ANOVA with post-hoc Tukey’s test. (**e**) At the indicated timepoint, NETs formation was assessed fluorometrically using SYTOX green after detachment of DNA with MNase, n = 6, 1-way ANOVA with posthoc Bonferroni’s test.

Visual inspection of live images confirmed that NEi inhibited peroxynitrite-induced NETs release (Figure 3a). In peroxynitrite-stimulated samples, after 2 h of stimulation, we detected mostly NET-releasing or decondensed cells, whilst preincubation with NEi noticeably inhibited NETs formation and resulted in the predominance of small, condensed, double-positive objects. Importantly, NEi showed no signs of cytotoxicity in unstimulated samples and control neutrophils pre-incubated with NEi preserved their multilobulated shape (Figure 3a). No effect of NEi on the morphology of ustimulated cells and inhibition of peroxynitrite-induced NETs formation by NEi could also be observed in immunofluorescently-labeled samples (Figure 3b). Notably, in samples preincubated with NEi and stimulated with peroxynitrite large proportion of neutrophils’ nuclei lost their multilobulated shape, but no signs of decondensation were evident (Figure 3b). Analyses performed with the pre-trained CNN model remained in perfect agreement with aforementioned live microscopy observations. Vast majority (≥94%) of objects in negative control incubated with or without NEi were classified as unstimulated cells, most objects in peroxynitrite-stimulated samples were classified as decondensed or NET-releasing cells (19.3 ± 3.1% and 72.4 ± 3.7% of objects, respectively) and over 70% of objects were classified as dead cells in samples preincubated with NEi and further stimulated with peroxynitrite (Figure 3c). Statistical analysis confirmed significant differences in NETs formation between samples stimulated with peroxynitrite and samples preincubated with NEi and then stimulated with peroxynitrite (Figure 3d). On the other hand, simultaneous spectrofluorometrical measurements of DNA release failed to show differences in NETs formation between samples pre-incubated with or without NEi and stimulated with peroxynitrite (Figure 3e). There was only a slight, insignificant reduction in DNA release in samples preincubated with NEi and stimulated with peroxynitrite compared to samples not treated with this inhibitor. Overall, these results confirmed a good performance of our model, superior to DNA release measurement-based assay and its utility to readily assess NETs formation using live-cell imaging.

## 4. Discussion

This work contributes considerably to the research on the neutrophil extracellular traps by proposing a breakthrough approach in identifying and quantifying NETs formation based on live imaging and processing of these images with a CNN-trained model. We provide evidence that this robust model achieves near-human performance and is superior to NETs quantification based on the measurement of extracellular DNA release. Moreover, this technology of data acquisition allows to analyze hundreds of images in much shorter time that manual counting of NETs in microscopical images and assures reproducibility of the results. In the proposed method, staining of the samples is performed using readily available DNA-binding dyes and the image acquisition procedure does not involve the use of expensive laboratory equipment like a confocal microscope—Images can be taken with an inverted fluorescence microscope. Additionally, analysis of images in the proposed approach can be carried out on a standard computer.

Soon after its discovery, the process of NETs formation turned out to be implicated in multiple pathologies [47,48,49,50]. Accordingly, it is predicted that quantification of NETs-associated markers could provide clinically useful information [51]. If NETs are to become a biomarker supporting patients management, the assay procedure and detection methods must be standardized across clinical laboratories. Moreover, to compare the results of various research studies, NET-specific measures need to be unified. To date, experimental conditions used by different authors vary between laboratories and these differences concern e.g., the type of cell culture medium used, addition of fetal bovine serum or albumin, material of which the chambers are made (plastic/glass), seeding densities, time of stimulation or concentrations of stimuli. Notably, all of the aforementioned variables can influence NETs formation [9,10,17,52,53]. Furthermore, there are multiple methodologies used to quantitatively assess the process of NETs formation–some of them relying on the measurement of the extracellular DNA using DNA-intercalating dyes, some of them using ELISA measurements of DNA complexed with neutrophil-derived proteins, and others are based on the analysis of microscopical images with manual or automated counting [10,54,55,56]. Even though a number of automated or semi-automated methodologies to quantify NETs based on microscopy images have been proposed [11,14,20], some of them relatively easy to implement on a large scale [12,13,17], still very few of them have been widely adopted by other researchers. Recently, an imaging flow cytometry has been proposed as a method for automatic determination of NETosis [21,57]. However, due to limited access of vast majority of laboratories to such an equipment, it is not likely to become widespread in the nearest future. The main advantages of the model we propose is the ease of use once the model has been trained and the ability to differentiate between SYTOX-positive NETs and SYTOX-positive cells of condensed chromatin, here designated as dead cells. 

In our hands, live-imaging-based CNN model was able to show inhibitory effect of NEi on peroxynitrite-induced NETs release, contrary to spectrofluorometric method, previously criticised due to inability to differentiate between different modes of cell death as a source of DNA release [10]. Importantly, simultaneous use of Hoechst 33342 and SYTOX Green for live imaging performed better than immunofluorescent labelling. Live imaging revealed the preponderance of dead cells in samples pretreated with NEi and stimulated with peroxynitrite, contrary to the dominance of unstimulated cells in untreated samples (negative controls). On the other hand, immunofluorescence labelling revealed only subtle alterations of nuclear morphology in both above-mentioned experimental set-ups, making these inconspicuous changes easy to overlook or misinterpret. What is more, simple immune processing of the samples would not allow to differentiate, whether changes in the nucleus morphology coincide with the disruption of plasma membrane. Live imaging of cells appears to be a good alternative to immune labelling, since it requires less processing and entails much lower risk of introducing artifacts [13,17,19]. However, immune-histological staining may be necessary to confirm the presence of NETs if uncommon inducers are used or in samples consisting of mixed cell populations (not solely isolated granulocytes). One should also bear in mind that NETs composition may differ and not all of the markers commonly used to identify NETs might be detected on all immunofluorescently-labeled NET-like structures [13].

Notably, although our study is not the first one to explore the potential of live imaging to analyze NETs formation process [17,19], this is the first approach utilizing CNN-based machine learning but not threshold-based algorithms. Even though an asset of the previously proposed methodology is higher interpretability due to its simplicity, a CNN-based model streamlines an image-analysis process (images do not require any pre-processing), as well as may take advantage of higher robustness towards imaging artefacts. Another advancement proposed by our study, in comparison with previous reports [17,19], is the ability to discriminate between resting, polymorphonuclear granulocytes (here designated as “unstimulated” cells) and rounded, decondensed, SYTOX Green-negative cells (here designated as “decondensed” cells). Changes of nuclear morphology and loss of the nuclear lobules is well-recognized as an early event preceding NETs release. With this additional class of images, NETs release process can be more thoroughly monitored at the early steps of its advancement.

In our study, we have decided to calculate standard performance metrics and also take into consideration those at very low IoU level, thus accepting higher localization errors. This decision came out from a belief, expressed also by Coelho et al. [14], that, for the purpose of NETs quantification, the most important is the correct biological result but not pixel correctness. Yet surprisingly the model’s performance at “standard: IoU = 0.5 is only slightly lower when compared with IoU = 0.1, which means that the model already achieved acceptable localization correctness.

The evidence has accumulated that the individual variations in enumeration of NETs-producing cells between observers may be the source of bias and/or systematic error [13,14]. Thus, the fact that our work capitalized on microscopic data for training of the CNN model provided by a human operator might be considered as a limitation of our study. The bias can occur if the method is based on human labelling of images implemented in the training dataset, but on the other hand, the model learns from the operator to differentiate between separate objects within conglomerate of several objects (Figure 2), just as well as a qualified operator can do. It was impossible or extremely difficult to reliably quantify NET-releasing objects among several adjacent cells using previous methods based on object size [12,13,19]; our model overcomes these limitations. The performance of the proposed model might be further improved by the enlargement of the training dataset, preferably prepared by a group of highly qualified scientists and by using a wide variety of stimuli of NETs release to include less typical morphological features of NET-releasing cells, such as blebbing of the nucleus [21]. Consistently, the results presented in Table 2 should be treated as a baseline for other researchers who will hopefully make use of the dataset that we publish along with this study.

## 5. Conclusions

To our knowledge, no study to date proposed open dataset for training of image analysis models to quantify the phenomenon of NETs release basing on a live-imaging technique. We have demonstrated that a modern CNN model outperforms quantification method based on the measurement of DNA release. The proposed approach does not require expensive, sophisticated equipment and the images can be analyzed with a public-domain software package. Moreover, further tuning of the algorithms for image analysis can improve the model. We hope that our model will contribute to concerted human effort to quantify NETs both for research and diagnostic purpose.

## Figures and Tables

**Table 1:** Dataset created using live imaging of neutrophils releasing NETs.

	Subset	Images	Cell Type			
			Unstimulated	Decondensed	NET	Dead
Split	Total	305	3017	638	1919	581
	Train	188	1918	492	1241	193
	Val	60	701	69	374	182
	Test	57	398	77	304	206

**Table 2:** Performance metrics of the adopted Convolutional Neural Network (CNN) model on our test subset.

Metric	Area			
	Small	Medium	Large	All
AP @ IoU = 0.50:0.95 (MS CoCo)	0.380	0.593	0.213	**0.593**
AP @ IoU = 0.50 (Pascal VOC)	0.580	0.930	0.305	**0.906**
AP @ IoU = 0.10	0.619	0.930	0.316	**0.913**
AR @ IoU 0.50:0.95	0.467	0.673	0.235	**0.666**
AR @ IoU = 0.50	0.625	0.946	0.317	**0.925**
AR @ IoU = 0.10	0.656	0.947	0.325	**0.931**

## Supplementary Materials

The following are available online at https://www.mdpi.com/2073-4409/9/2/508/s1, Figure S1. Neutrophil elastase inhibitor diminishes NETs formation upon phorbol 12-myristate 13-acetate (PMA), *S*-nitroso-*N*-acetyl-DL-penicillamine (SNAP), and peroxynitrite stimulation; Figure S2. Peroxynitrite but not SNAP induce NETs release by neutrophils isolated from chronic granulomatous disease (CGD) patients; Figure S3. Examples of NETs-objects incorrectly classified by the pre-trained CNN model.
