# Supplementary material for: Convolutional Neural Networks–Based Image Analysis for the Detection and Quantification of Neutrophil Extracellular Traps
Source: Cells. 2020 Feb 24;9(2):508. doi: 10.3390/cells9020508 (PMC7072771; doi:10.3390/cells9020508)
Supplement: Supplementary file 1 [file cells-09-00508-s001.pdf]

Supplementary data – Convolutional neural networks-based image analysis for detection and quantification of neutrophil extracellular traps

Supplementary Materials and Methods

Model training

The list of hyper-parameters and training parameters adopted in our work with Mask R-CNN model implemented by [1] is provided below:

```
# minimal confidence of the model allowing to mark a detected object
DETECTION_MIN_CONFIDENCE = 0.5

# non-maximum suppression threshold
DETECTION_NMS_THRESHOLD = 0.15

# number of images fed to a single GPU
IMAGES_PER_GPU = 1

# number of used GPUs
GPU_COUNT = 1

# applied learning rate and learning momentum
LEARNING_RATE = 0.0001
LEARNING_MOMENTUM = 0.9

# number of training epochs and steps per epoch
EPOCHS = 150
STEPS_PER_EPOCH = 40

# model layers chosen for training
LAYERS = ALL

 # Number of validation steps to run at the end of every training epoch.
 VALIDATION_STEPS = 2

# "Backbone" model architecture
BACKBONE = "resnet101"

# The strides of each layer of the Feature Pyramid Network
BACKBONE_STRIDES = [4, 8, 16, 32, 64]

# Size of the fully-connected layers in the classification graph
FPN_CLASSIF_FC_LAYERS_SIZE = 1024

# Size of the top-down layers used to build the feature pyramid
TOP_DOWN_PYRAMID_SIZE = 256

# Number of classification classes (including background)
NUM_CLASSES = 5

# Length of square anchor side in pixels
RPN_ANCHOR_SCALES = (32, 64, 128, 256, 512)

# Ratios of anchors at each cell (width/height). A value of 1 represents a square anchor, and 0.5 is a wide anchor
RPN_ANCHOR_RATIOS = [0.5, 1, 2]
```

```
# Anchor stride: if 1 then anchors are created for each cell in the backbone feature map, if 2, then anchors are created for every other cell, and so on.
RPN_ANCHOR_STRIDE = 1

# Non-max suppression threshold to filter Region Proposal Network proposals
RPN_NMS_THRESHOLD = 0.7

# How many anchors per image to use for Region Proposal Network training
RPN_TRAIN_ANCHORS_PER_IMAGE = 256

# ROIs kept after non-maximum suppression (training and inference)
POST_NMS_ROIS_TRAINING = 2000
POST_NMS_ROIS_INFERENCE = 1000

# Resizes instance masks to a smaller size to reduce memory load, (height, width) of the mini-mask
USE_MINI_MASK = True
MINI_MASK_SHAPE = (7, 7)

    # Input image resizing
    # square: Resize and pad with zeros to get a square image
    IMAGE_RESIZE_MODE = "square"
    IMAGE_MIN_DIM = 700
    IMAGE_MAX_DIM = 1024

    # Image mean (RGB)
    MEAN_PIXEL = np.array([123.7, 116.8, 103.9])

    # Number of ROIs per image to feed to classifier/mask heads
    # The Mask RCNN paper uses 512 but often the RPN doesn't generate
    # enough positive proposals to fill this and keep a positive:negative
    # ratio of 1:3. You can increase the number of proposals by adjusting
    # the RPN NMS threshold.
    TRAIN_ROIS_PER_IMAGE = 200

    # Percent of positive ROIs used to train classifier/mask heads
    ROI_POSITIVE_RATIO = 0.33

    # Pooled ROIs
    POOL_SIZE = 7
    MASK_POOL_SIZE = 14

    # Shape of output mask
    MASK_SHAPE = [28, 28]

    # Maximum number of ground truth instances to use in one image
    MAX_GT_INSTANCES = 100

    # Bounding box refinement standard deviation for RPN and final detections.
    RPN_BBOX_STD_DEV = np.array([0.1, 0.1, 0.2, 0.2])
    BBOX_STD_DEV = np.array([0.1, 0.1, 0.2, 0.2])

    # Max number of final detections
    DETECTION_MAX_INSTANCES = 100

    # Weight decay regularization
    WEIGHT_DECAY = 0.0001
```

```
# Loss weights for more precise optimization.
# Can be used for R-CNN training setup.
LOSS_WEIGHTS = {
    "rpn_class_loss": 1.,
    "rpn_bbox_loss": 1.,
    "mrcnn_class_loss": 1.,
    "mrcnn_bbox_loss": 1.,
    "mrcnn_mask_loss": 1.
}

# Use RPN ROIs or externally generated ROIs for training
USE_RPN_ROIS = True

# Train or freeze batch normalization layers
#     None: Train BN layers. This is the normal mode
#     False: Freeze BN layers. Good when using a small batch size
#     True: (don't use). Set layer in training mode even when inferencing
TRAIN_BN = False

# Gradient norm clipping
GRADIENT_CLIP_NORM = 5.0
```

When training our Mask-R CNN-based model, we did not perform any class balancing, since it is difficult to predict the "natural" distribution of classes – it may sharply rely on the experimental setting, e.g. the use of specific inducers and inhibitors of NETs formation.

Supplementary Figures

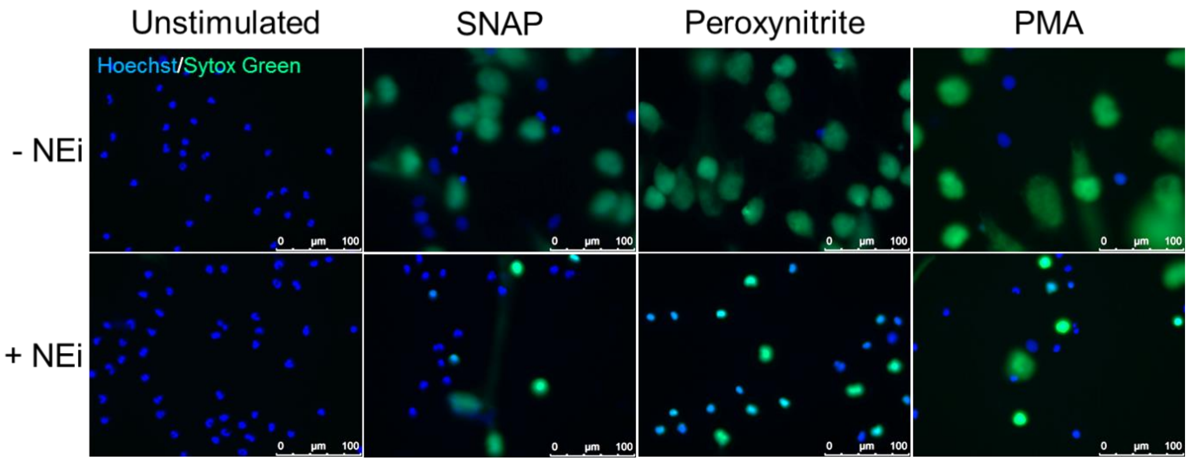

Figure S1. Neutrophil elastase inhibitor diminishes NETs formation upon phorbol 12-myristate 13-acetate (PMA), S-nitroso-N-acetyl-DL-penicillamine (SNAP) and peroxynitrite stimulation. Isolated human neutrophils were seeded into plates, pre-incubated with neutrophil elastase inhibitor (NEi) for 30 min when necessary and stimulated with PMA, SNAP or peroxynitrite or left unstimulated. After 3-hour incubation cells were simultaneously stained with Hoechst 33342 and SYTOX Green. Samples were visualized with inverted fluorescent microscope at magnification 40×. Representative images out of six independent experiments using different donors are shown.

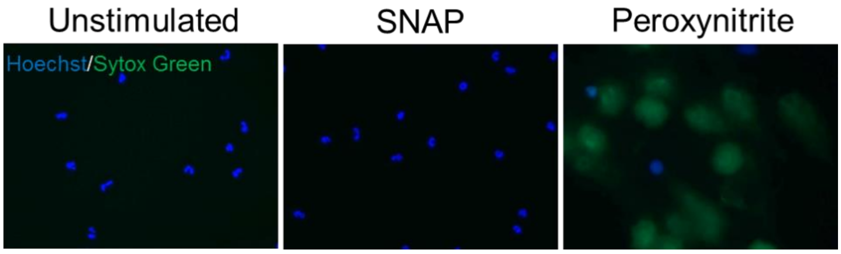

Figure S2. Peroxynitrite but not SNAP induce NETs release by neutrophils isolated from chronic granulomatous disease (CGD) patients. Neutrophils were isolated from patients suffering from CGD, seeded into plates, allowed to settle for 30 min and stimulated with SNAP, peroxynitrite or left unstimulated. After 3-hour incubation cells were simultaneously stained with Hoechst 33342 and SYTOX Green. Samples were visualized with inverted fluorescent microscope at magnification 40×. Representative images out of seven independent experiments using different donors are shown.

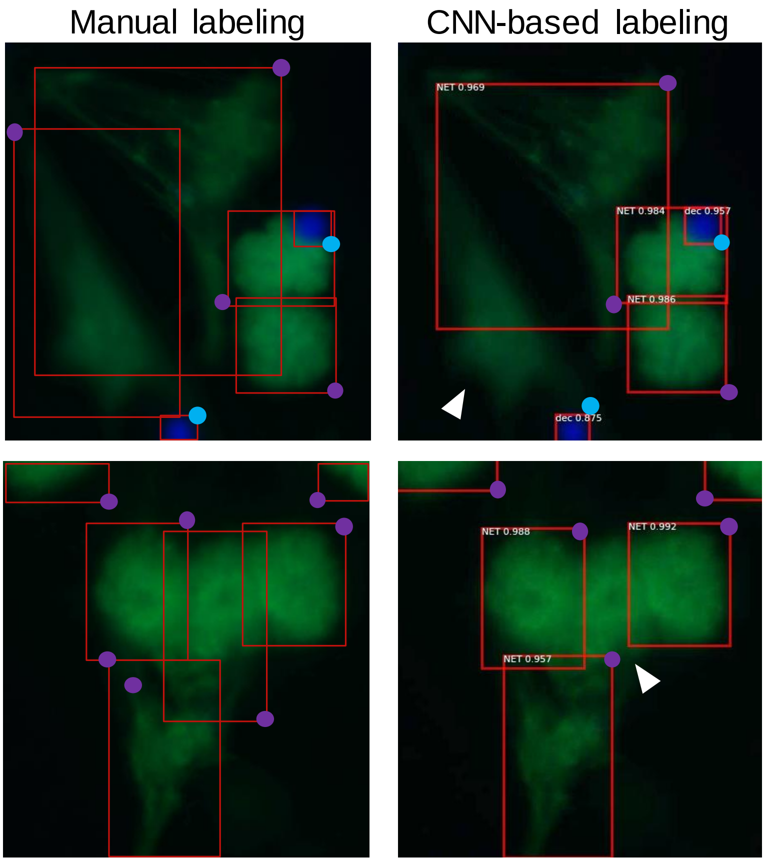

Figure S3. Examples of NETs-objects incorrectly classified by the pre-trained CNN model. Images of unfixed neutrophils stained with Hoechst 33342 (blue) and SYTOX Green (green), analyzed by the CNN model (right) in parallel with manual analysis (left) are shown. To facilitate the assessment of labeling, colored dots are shown at one of the four vertexes of a rectangle surrounding the object; blue dots – decondensed cells (dec), violet dots – NETs. Numerical values represent model's confidence in the given cell class prediction – 1 is maximum confidence. White arrows indicate objects missed with an automatic, CNN-based labelling.

References

1. Abdulla, W. Mask R-CNN for object detection and instance segmentation on keras and tensorflow. Preprint at https://github.com/matterport/Mask_RCNN 2017.
